# Cloning and expression of *Saccharomyces cerevisiae SUC2* gene in yeast platform and characterization of recombinant enzyme biochemical properties

**DOI:** 10.1007/s13205-016-0441-7

**Published:** 2016-06-08

**Authors:** Nooshin Mohandesi, Seyed Omid Ranaei Siadat, Kamahldin Haghbeen, Ardeshir Hesampour

**Affiliations:** 1 National Institute of Genetic Engineering and Biotechnology (NIGEB), Tehran, Islamic Republic of Iran; 2 Protein Research Centre, Shahid Beheshti University, GC, Tehran, Islamic Republic of Iran; 3 Department of Biology, Central Tehran Branch, Islamic Azad University, Tehran, Islamic Republic of Iran

**Keywords:** *Saccharomyces cerevisiae*, Recombinant invertase, *Pichia pastoris*, *SUC2* gene, Cloning

## Abstract

Invertase (EC.3.2.1.26) catalyzes the hydrolysis of sucrose to an equimolar mixture of d-glucose and d-fructose which is of interest for various industrial applications. In this research, *Saccharomyces cerevisiae* invertase gene (SUC2) was optimized based on *Pichia pastoris* codon preference. The synthetic gene was introduced into the methylotrophic yeast *Pichia pastoris* under the control of the inducible AOX1 promoter. High level of the extracellular recombinant invertase (R-inv) production was achieved via methanol induction for 4 days and purified by His-Tag affinity chromatography which appeared to be a mixture of glycosylated proteins with various sizes of 85–95 kDa on SDS-PAGE. Deglycosylation of the proteins by Endo-H resulted in the proteins with average molecular weight of 60 kDa. The purified recombinant invertase biochemical properties and kinetic parameters determined a pH and temperature optimum at 4.8 and 60 °C, respectively, which in comparison with native *S. cerevisiae* invertase, thermal stability of recombinant invertase is highly increased in different heating treatment experiments. The purification of recombinant invertase resulted in an enzyme with specific activity of 178.56 U/mg with 3.83-fold of purification and the kinetic constants for enzyme were Km value of 19 mM and Vmax value of 300 μmol min^−1^ mg^−1^ With kinetic efficiency (Kcat/Km) of 13.15 s^−1^ mmol^−1^ it can be concluded that recombinant *P. pastoris* invertase can be more effective for industrial quality criteria. We conclude that recombinant *P. pastoris* enzyme with broad pH stability, substrate specificity and proper thermal stability can fulfil a series of predefined industrial quality criteria to be used in food, pharmaceutical and bio ethanol production industries.

## Introduction

Invertases [β-d-fructofuranoside fructohydrolase (EC 3.2.1.26)] are disaccharidases which belong to the Gh32 family of glycoside hydrolases and catalyse the hydrolysis of sucrose into an equimolar mixture of d-glucose and d-fructose. To produce invert sugar at concentrations lower than 10 % sucrose that are more stable than pure sucrose syrups and with less carbohydrate are sweeter than sucrose and minimise crystallisation in comparison with sucrose at high (Gehlawat 2001; Boer et al. 2004; Mona et al. 2009; Patil et al. 2012) concentration. They have been reported to be produced by variety of higher plants, some animal cells and wide range of microorganisms including bacteria and fungi (Kim et al. 2000).

The Production of invert sugar and high fructose syrup from sucrose by acid hydrolysis method is uneconomic, because of its low conversion efficiency (65–70 %) and formation of undesirable products (7–8 %) (Kaur and Sharma 2005; Shankar et al. 2014). Therefore, biological sources due to their easy cultivation, high yields of producing enzyme and ability to hydrolyse sucrose by a single step are remarkable alternatives.

Preparation of invert sugar and high fructose syrup by acting on broad cost effective substrate spectrum such as sucrose, sugarcane bagasse, molasses and grape juice residue making invertase suitable for biotechnological applications and due to its interesting functional properties (Kaur and Sharma 2005; Gracida-Rodriguez et al. 2006; Mona et al. 2009; Driouch et al. 2010), the market potential of invertases includes applications in food and dairy industries, pharmaceutical, cosmetic, paper and tobacco industries, production of confectionary with liquid center, bakery, calf feed preparation and fermentation of cane molasses into ethanol. Also, increasing disturbances on pollution that occurs from industrial wastes, has encouraged interest in converting waste materials to useful products using invertase (Gehlawat 2001; Kaur and Sharma 2005; Gracida-Rodriguez et al. 2006; Mona et al. 2009; Driouch et al. 2010; Uma et al. 2010a, b; Shankar et al. 2014). Invertase production from native sources cannot persuade the increasing market demand due to low yields which is not compatible with the industrial fermentation processes (Cereghino and Cregg 2000; Piscitelli et al. 2010). Recombinant protein expression in cost effective cultivable and handling host can provide higher productivity in short period of time with low costs. Regarding this effect on industry, increasing the level of invertase expression is considerable. Despite the success in using *Saccharomyces cerevisiae* as a host for expression of eukaryotic genes, some problems like hyperglycosylation, less secretion of recombinant protein and low level of expression reduce its potential in biotechnological industry. For these reasons, instead of *S. cerevisiae*, methylotrophic yeast systems such as *Pichia pastoris* because of correct glycosylation profile, cost effective bulk recombinant protein production, high level expression, secretion of heterologous protein to media and easy scale up expression which is important for biotechnological industry, have been used (Cereghino and Cregg 2000). The objective of this work was to investigate the expression and extracellular production of recombinant invertase of optimized *SUC2* gene from the source of *S. cerevisiae* which was expressed in methylotrophic yeast *P. pastoris.* Expression of the optimized *SUC2* gene in *P. pastoris* yields a secreted active recombinant protein with a little different molecular weight to the invertase secreted by *S. cerevisiae*, after purification of enzyme, biochemical properties and kinetic parameters of purified recombinant invertase was determined.

## Materials and methods

### Strains, plasmid, growth conditions

*Escherichia coli* strain DH5α was used for bacterial transformation and plasmid propagation. *E. coli* strain DH5α was grown in Luria–Bertani (LB) medium [(1 % (w/v) yeast extract, 2 % (w/v) peptone, 1 % (w/v) NaCl] at 37 °C and supplemented with ampicillin (100 μg/ml) for Screening of Transformants.

*Pichia pastoris* strain 4 was employed as a protein expression host in this work and expression vector pPinkα-HC (7.9 kb) were obtained from Invitrogen, (San Diego, CA). Yeasts were cultivated in Yeast extract Peptone Dextrose (YPD) medium (1 % yeast extract, 2 % peptone, 2 % glucose at 30 °C (Table 1). For expression of recombinant enzyme, transformed *P. pastoris* was incubated in buffered glycerol-complex (BMGY) medium (0.1 M sodium phosphate pH 6.0, 1 % (w/v) yeast extract, 2 % (w/v) peptone, 1.34 % (w/v) yeast nitrogen base (YNB), 0.00004 % (w/v) biotin, and 1 % (v/v) glycerol) at 30 °C in shaker at 200 rpm. For induction of invertase gene expression by methanol was used by buffered methanol-complex (BMMY) medium [0.1 M sodium phosphate pH 6.0, 1 % (w/v) yeast extract, 2 % (w/v) peptone, 1.34 % (w/v) YNB, 0.00,004 % (w/v) biotin, and 0.5 % (v/v) methanol].

**Table 1:** Strains, plasmids and synthetic oligonucleotide primers

Item	Description	Reference
Strains		
*E. coli* DH5α	*E. coli*, α-complementation	Fermentase (USA)
*Pichia pastoris*	Protein expression host	Invitrogen (USA)
*Saccharomyces cerevisiae*	Native Inveratse source	Sigma (USA)
Plasmid		
pPinkα-HC	For integration in *P. pastoris*	Invitrogen (USA)
Oligonucleotide primers		
INV-P Forward	5′-TCTATGACTAACGAAACTTCTG-3′	
INV-P Reverse	5′-CTTAACTTCTCTAACTTGGAAC-3′	
5′ Aox1 primer	5′-GACTGGTTCCAATTGACAAGC-3′	
3′ Aox1 primer	5′-GCAAATGGCATTCTGACATCC-3′	

### Chemical and enzymes

DNA restriction enzymes and T4 DNA Ligase were purchased from Vivantis Technologies Co. (Malaysia). Taq DNA polymerase, DNA Ladders and Protein markers were from Fermentase Thermo Fermentase (USA). Endoglycosidase H (Endo H) was purchased from New England Biolabs (Beverly, MA, USA).

Native invertase was purchased from Sigma-Aldrich (USA) for using as positive control enzyme in kinetic experiments. Oligonucleotides were synthesized at TAD Copenhagen A/S (Denmark). Sucrose was from Merck (Darmstadt, Germany) and other chemicals were obtained from Merck (Darmstadt, Germany) or Sigma Chemical Co. (St Louis, MO, USA).

### Construction of invertase expression vector

The *SUC2* gene of *S. cerevisiae*, encoding invertase was designed according to codon preference of *P. pastoris* excluding the sequence of the N-terminal pre-pro-sequence signal peptide and His-tag in the C terminus for enabling purification of the expressed invertase directly from growth medium by affinity chromatography. Designed gene (Gene Bank Accession No. CAA87030) was synthesized by Gene Art Company (Germany).

The synthetic *SUC2* gene in PUC57 vector was transformed to *E. coli* strain DH5α and transformants were screened in LB media supplemented with ampicillin (100 μg/ml).

**Fig. 1:**
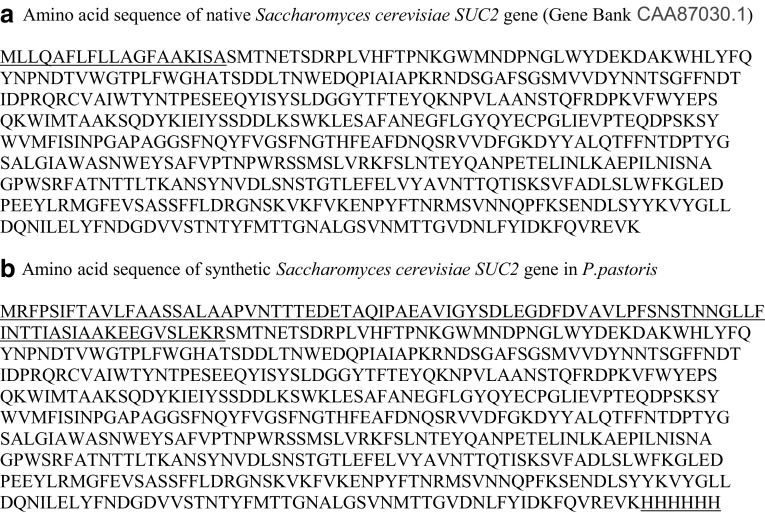
Invertase Amino acid sequence. **a** The amino acid sequence of native protein and its N-terminal native signal peptide (*Underlined*). **b** The amino acid sequence of synthetic invertase protein of *P. pastoris* (N-terminal alpha mating factor signal peptide of *S. cerevisiae and His tag in the C*-*terminal* are *underlined*)

After performing colony PCR for confirmation of the transformed clones containing PUC-*SUC2*, with specific forward (INV-F) and reverse (INV-R) primers (Table 1). Plasmid extraction was carried out by GF1 Nucleic Acid extraction kit (Vivantis Technologies Co. Malaysia) and digested with *KpnI/XhoI*. The digested product was separated by 1 % agarose gel electrophoresis and gel slice containing the expected size band (1550 bp) was excised and ligated into pPinkα-HC, expression vector contained the pro-pre-sequence of *S.cervisiae* α mating factor under the control of methanol-inducible alcohol oxidase (AOX1) promoter for expressing the inserted foreign gene in yeast (Fig. 1) which had been digested with the same restriction enzymes. The ligated product was used to transform *E. coli* DH5α competent cells, and the transformed cells were plated on LB media with ampicillin. The correct transformants were checked By PCR. The PCR conditions were: 95 °C for 7 min for one cycle and then continued by 95 °C for 1 min, 55 °C for 40 s and 72 °C for 35 s for 30 cycles. The Sequence of PCR product was confirmed by DNA sequencing (Applied Biosystem, ABI, CA, USA). Finally, the recombinant vector was obtained, it was later confirmed by sequencing with AOX primers (Table 1). DNA manipulation was performed according to the standard procedure (Sambrook et al. 1989).

### Expression of recombinant enzyme

For transformation to *P. pastoris*, 1 μg of pPinkα-SUC2 recombinant expression vector was linearized using *Vha 464I* at 37 °C for 2 h, after which linearized plasmids were purified by GF1 Nucleic Acid extraction kit (Vivantis Technologies Co. Malaysia) which was according to the manufacturer’s protocol. For the expression of recombinant protein, the competent adenine gene deficient *P. pastoris* was prepared and transformed with the plasmid pPinkα-HC using the electroporation method (BTX, ECM630, USA) at 1850 V, 200 Ω and 25 µF with 2 mm cuvette. Immediately, after pulsing 1 ml of cold 1 M sorbitol was added to the sample and incubated at 30 °C for 60 min, then cuvette contents were plated on PAD medium (*Pichia* Adenine Dropout). The plates were incubated for 3–7 days at 30 °C, until distinct colonies are formed. The linearized pPinkα-HC was transformed to *P. pastoris* as a negative control.

### Screening of transformed yeasts

Integration of *SUC2* to yeast host genomes was investigated by extraction of genomic DNA of transformed *P. pastoris* which was grown on YPD medium. Yeasts genomic DNA were extracted by glass beads method as described by Hoffman and Winston (1987) and the presence of *SUC2* gene was confirmed by PCR using specific primers (Table 1). The PCR conditions were: 95 °C for 5 min for one cycle, then continued by 95 °C for 30 s, 55 °C for 35 s and 72 °C for 40 s for 30 cycles. The amplified PCR product was checked by 1 % agarose gel electrophoresis and the expected size band (1550 bp) was observed. The Sequence of amplified PCR product was confirmed by DNA sequencing (Applied Biosystem, ABI, CA, USA).

### Expression of recombinant invertase

Basically, the correct recombinant yeasts were cultured in BMGY medium at 30 °C in shaker incubator at 220 rpm until the *A*_600_ value reached 2.0. Culture samples were then centrifuged at 3000 *g* for 5 min, after which the growth medium was removed and the cells were resuspended in the BMMY medium and grown at 30 °C for 4 days. Methanol [(0.5 % (v/v) of the growth medium] was added to the culture every 24 h to induce invertase gene expression.

### Protein purification and detection

Purification of the recombinant invertase was carried out by affinity chromatography. The methanol-induced cultures were centrifuged at 5000*g* for 5 min, after which the invertase-containing supernatant was lyophilized and resuspended dry powder in Binding buffer [20 mM phosphate, 20 mM imidazole, 500 mM NaCl (pH 7.4)] and latter concentrated by ultrafiltration Amicon Millipore (30 kDa cut off).

The concentrated enzyme solution was pumped to AKTA Purifier Fast Protein Liquid Chromatography system (Amersham bioscience, USA) by applying a HisTrap FF Crude 5-ml column (GE Healthcare, USA) equilibrated with Binding buffer, and after sample injection, it was washed with 5 column volumes of Binding buffer, followed by 2.5 column volumes of 10 % Elution buffer [20 mM phosphate, 500 mM imidazole, 500 mM NaCl (pH 7.4)]. The enzyme of interest was eluted with a linear gradient from 10 to 100 % Elution buffer in 5 column volumes, and finally, the column was washed with 5 column volumes of 100 % Elution buffer (Lafraya et al. 2011). Then, the fractions with invertase activity were collected and 10 % SDS-PAGE was performed according to the method of Laemmli (Laemmli 1970) for resulting fractions and stained by silver nitrate. Sample containing a band with the expected size of enzyme was dialyzed (1/10,000) against a buffer containing 50 mM Sodium acetate (pH 4.8) and kept at −20 °C for further analysis.

### Determination of Invertase activity

Invertase activity was assayed by 20 min incubation of 0.1 ml (1:10) diluted enzyme solution with 0.9 ml of sucrose in 50 mM sodium acetate buffer (pH 4.8) at 50 °C. Reducing sugars were quantified by addition of dinitrosalicylic acid reagent and the reaction was terminated by heating in 100 °C boiling water bath for 10 min. Finally, absorbance was read at 540 nm in spectrophotometer (Sainz-Polo et al. 2013). One unit of invertase was defined as the amount of enzyme that catalyzed the formation of l μmol of reducing sugar/min from sucrose at 50 °C and pH 4.8. The standard curve was prepared with glucose (0.25–2.5 mM).

The protein concentration was determined spectrophotometrically using an adsorption at 280 nm according to the method proposed by Gill and von Hippel (Gill and von Hippel 1989) with bovine serum albumin serving as the standard protein.

Enzymatic deglycosylation of the recombinant invertase was accomplished by incubating 5 μg of the invertase with 0.5 units Endo H (Biolab) for 18 h at 37 °C according to manufacturer instruction, invertase band migration shift was studied by SDS-PAGE. After which the resolved proteins were obtained with Coomassie blue R-250.

### Characterization of the purified recombinant enzyme

#### Effect of pH on activity and stability of the recombinant invertase

The effect of pH on invertase activity was determined in the pH range of 3–8, using 50 mM sodium acetate buffer (pH 3.0–5.0), sodium phosphate (6.0–7.0) and Tris–Hcl (8.0) and enzyme activity was measured after 20 min in 50 °C. To study the effect of pH on the stability, the enzyme was incubated for 30 min at 30 °C in 50 mM buffers at different pH in the absence of substrate, and after which calculated the residual activity under assay condition.

#### Effect of temperature on activity and stability of the recombinant invertase

The optimum temperature of invertase was measured in 50 mM sodium acetate buffer at pH 4.8 over a temperature range of 20 to 80 °C with interval of 5 °C. Thermal stability of enzyme was studied by incubating the purified enzyme samples in the absence of substrate at seven different incubation temperatures ranged from 20 to 80 °C for 1 h and immediately samples were placed on ice for 20 min, after which remaining activity of invertase measured under standard assay condition. Long time invertase thermal stability determined through 70 and 80 °C heat treatment from 1 to 60 min, subsequently invertase heat stability was determined according to samples residual activity.

The kinetic parameters, K_m_ and V_max_, Michaelis–Menten constants of invertase were determined at pH 4.8 with 50 mM sodium acetate buffer for five parallel series of recombinant invertase samples.

## Results and discussion

### Construction of vector for invertase expression in methylotrophic yeast

**Fig. 2:**
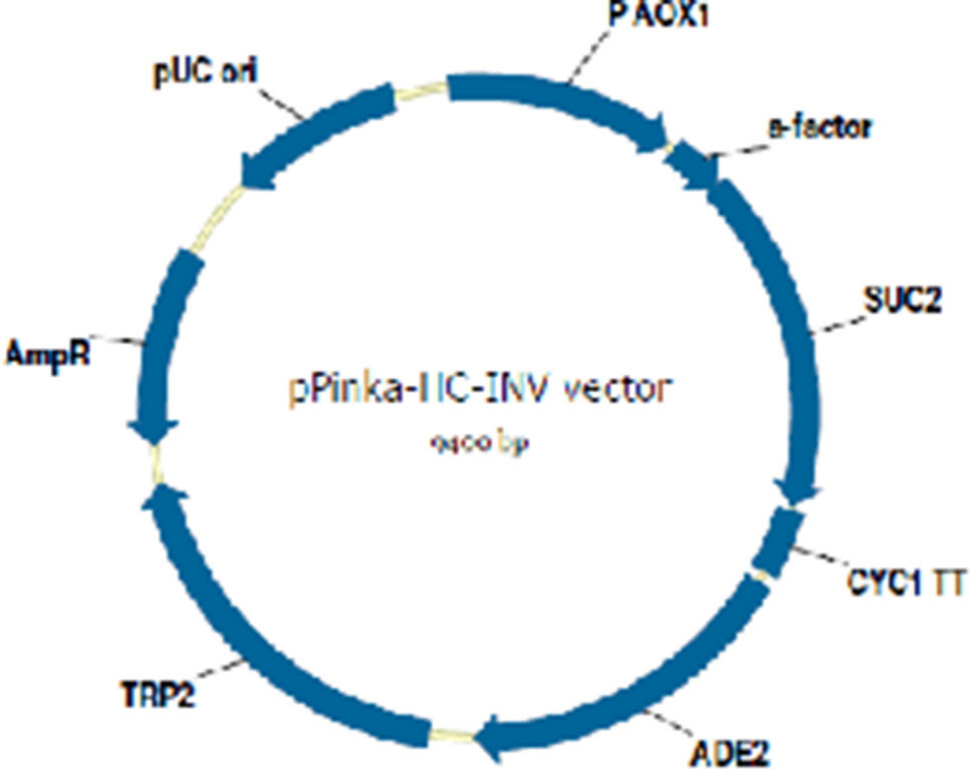
Construction of pPinkα-HC INV expression vector

The *SUC2* gene (1550 bps) with open reading frame encoding 512 amino acid residues and 6 histidine residues in C terminus that enable purification of the expressed proteins by affinity chromatography, was designed according to methylotrophic yeast *Pichia pastoris* codon bias and for the first time in studies designed gene was optimized by Gene Optimizer software (Fig. 1). In others studies optimized synthetic gene was used for producing extracellular enzymes like cellulase and phytase which were succeed to express recombinant proteins (Akbarzadeh et al. 2013; Hesampour et al. 2014, 2015). Design of 6 histidine residues were successfully applied at C-terminal of the protein and enabled us to purify active enzyme, so probably small His-Tag in C terminus of invertase had no influence on the protein conformation for its activity (Huang et al. 2003). The synthesized *SUC2* gene was digested and cloned into pPink-αHC expression vector, afterwhich for efficient and stable expression of invertase, the recombinant pPinkαHC–SUC2 construct was linearized by *Vha 464I* restriction enzyme digestion at TRP2 region of plasmid and used to integrate in *P. pastoris* genome by genome replacement. The pPinkαHC–SUC2 was linearized by *Vha 464I* restriction enzyme digestion at TRP2 region of plasmid and transformed to *P. pastoris* cells. pPink-αHC contains a methanol-inducible alcohol oxidase (AOX1) promoter for expression of the invertase gene in yeast and for extracellular invertase secretion, α-mating factor from *S. cerevisiae* was used in the N terminus of recombinant expression construct (Fig. 2). The *S. cerevisiae* α-mating factor pre-pro-sequence provide secretion of numerous heterologous expressed products in *S. cerevisiae* and *P. pastoris* (Huang et al. 2003; Wang et al. 2005).

**Fig. 3:**
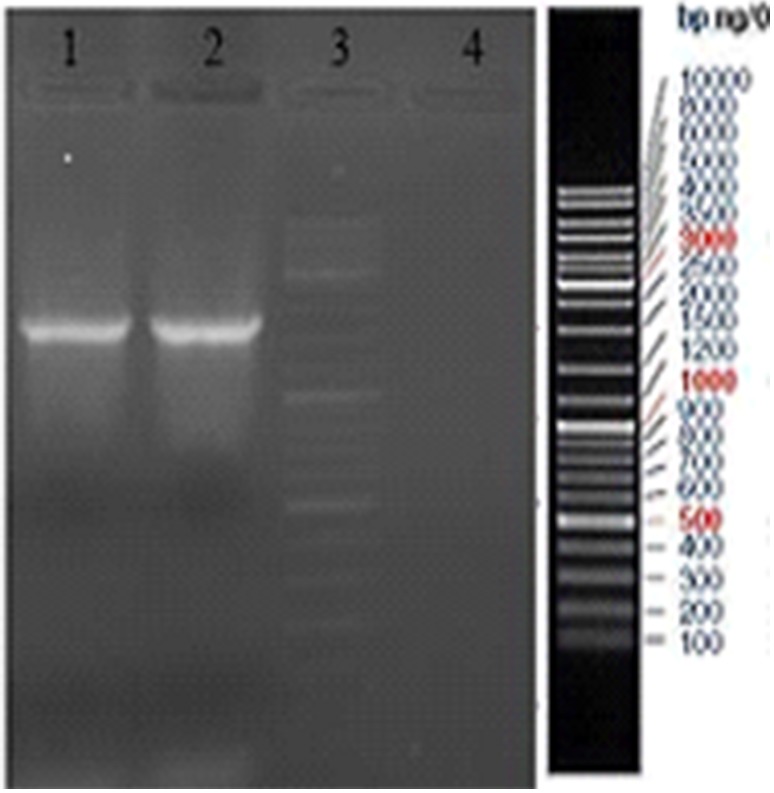
Yeast genome integrant PCR analysis. *Lane 1* PCR product of transformant *P. pastoris. Lane 2* pPink-INV plasmid PCR product as a positive control. *Lane 3* DNA size marker1 kb. *Lane 4* Transformed *P. pastoris* by pPinkα-HC Plasmid PCR as negative control

To confirm the correct *SUC2* gene integrated in yeasts genome, the genomes of yeasts which had been formed colonies in YPD media, extracted and screened by use of *SUC2* specific primers through PCR process. PCR product bands in 1 % agarose gel electrophoresis, revealed the successful genome integration (Fig. 3). In the next step, positive colonies were selected for subsequent studies.

### Expression of recombinant invertase in methylotrophic yeast

**Fig. 4:**
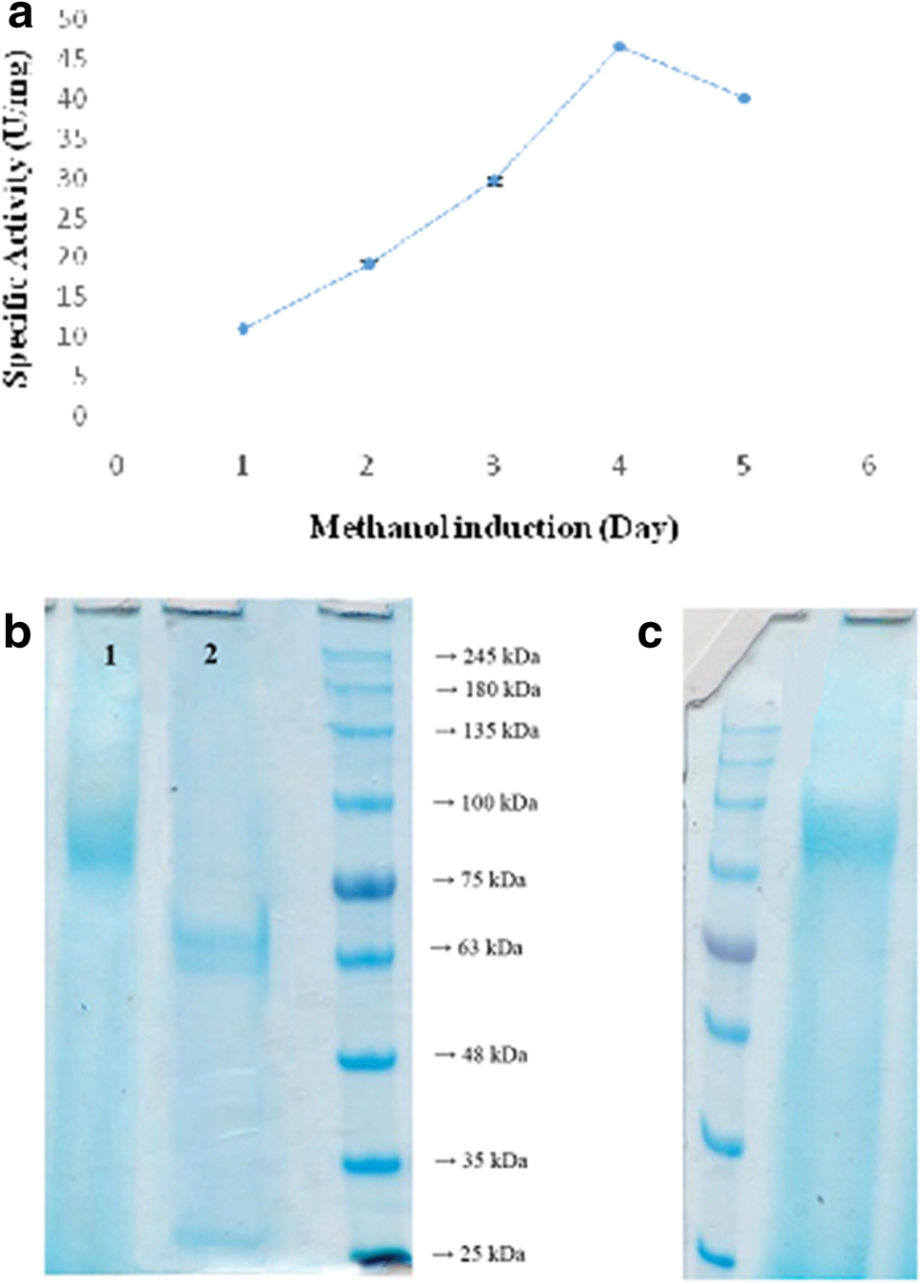
**a** Extracellular invertase activity by methanol induction. **b** SDS–Polyacrylamide gel electrophoresis of recombinant invertase from *P. pastoris*. *Lane 1* Recombinant expressed Invertase. *Lane 2* Recombinant expressed Invertase treatment with Endo H. **c** SDS-PAGE of native invertase from *S. cerevisiae*

Extra cellular Invertase production by transformed *Pichia* cells was studied in BMGY and BMMY expression media in the presence of methanol for induction of AOX1 promoter to overexpress the inserted invertase gene. Methanol was added to the culture every 24 h. Enzyme quantitative assays and SDS-PAGE were carried out after each day by methanol induction for determination of the highest invertase expression profile. Our results indicated that the recombinant active invertase expressed successfully in *P. pastoris* and secreted into the growth medium and as shown in Fig. 4a, it can be observed that extracellular invertase reached maximum activity after 4 days methanol induction (46.61 U/mg in 200 ml culture), followed by a decline in the enzyme activity till the 5th day of methanol induction. The results of *P. pastoris* supernatant SDS-PAGE showed that the smear bands with molecular size ranging from 85 to 95 kDa (Fig. 4b), whereas native invertase of *S*. *cerevisiae* with a 50 % carbohydrate content has a molecular mass of approximately 110–120 kDa that was included in the same gel (Fig. 4c) which in several studies were stated that invertase secreted by wild-type *Saccharomyces* strains consists of 100–140 kD (Trimble and Maley 1977) acid invertases have been shown to be glycosylated. Moreover, post-translational glycosylation is also involved in the secretion of heterologously expressed proteins from *P. pastoris*, though the glycosylation level and carbohydrate structures on the secreted recombinant proteins vary from protein to protein and this difference in molecular mass of enzymes can be concluded from the variant pattern of yeast glycosylation (Montesino et al. 1998). To determine the molecular size of distribution oligosaccharides associated with the recombinant invertase, the protein was deglycosylated by treatment with Endo H. SDS-PAGE analysis revealed that a shift in the apparent molecular mass to approximately 60 kDa according to its amino acid composition (Fig. 4b) and showed a similar electrophoretic mobility and molecular weight with the native *S. cerevisiae* invertase that was treated with Endo H by the same manner (Reddy et al. 1988; Perez et al. 2001). Translation of the recombinant invertase sequence produces a protein with 518 amino acids and molecular mass of close to 60 kDa, proposing the presence of N-linked carbohydrate contributed approximately 30–35 % for recombinant invertase of *P. pastoris* to the total weight of the enzyme.

### Effect of pH on invertase activity and stability

**Fig. 5:**
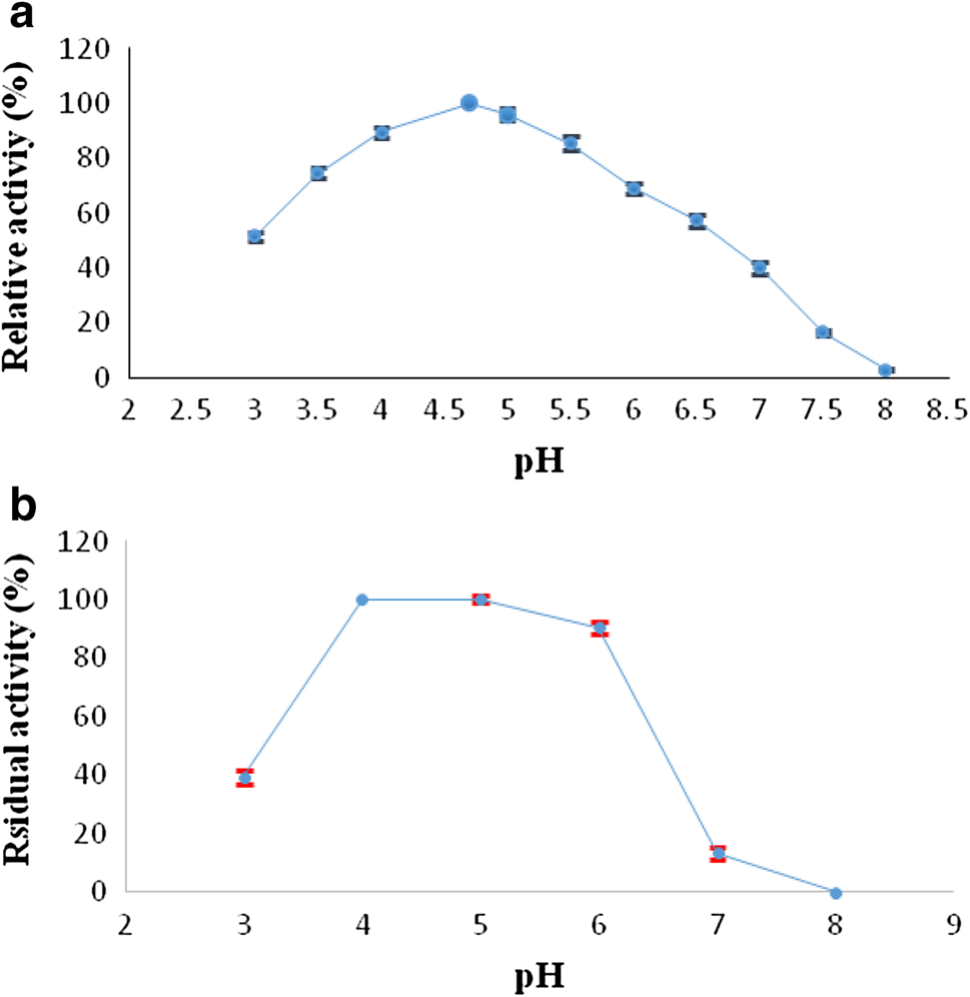
Characterization of recombinant Invertase expressed in *P. pastoris*, effect of different pH on recombinant Invertase assay. **a** Effect of different pH on recombinant Invertase activity. **b** Residual activity of recombinant Invertase after treatment for 30 min at different pH

The optimum pH invertase activity was evaluated by maintaining the enzyme in different pH reagent range of 3.0–8.0 (Fig. 5a). The results indicated that more than 50 % residual activity of enzyme in pH 3.0–6.5 with optimum activity at pH 4.8. Heterologous expressed invertase has exhibited the same pH pattern as the acid invertase and has potential to remain its activity in broad range of acidic to neutral environments that can be applied in bioethanol production, pharmaceutical industry and submerged fermentation. (Park and Sato 1982; Mona et al. 2009; Uma et al. 2010a; Patil et al. 2012).

For determination of long-term pH stability of the enzyme, the protein was placed at different pH in the absence of substrate. Extracellular invertase was found to be stable at pH 4.0–6.0 with more than 90 % residual activity after 30 min (Fig. 5b). The recombinant expressed invertase showed the acidic pH profile compared with native *S. cerevisiae* genus invertases which have recorded with optima pH 6.0 (Mona et al. 2009; Shankar et al. 2014) and also maximum *Aspergillu*s sp. Invertases activity were evaluated at pH 6.0 (Uma et al. 2010a, 2012; Patil et al. 2012) but similar pH profile observation have been reported for invertase production by recombinant *A.nigersuc1* gene (Driouch et al. 2010) and invertase -encoding of *AINV* gene *Arxula adeninivorans* (Boer et al. 2004).

### Effect of temperature on invertase activity and stability

**Fig. 6:**
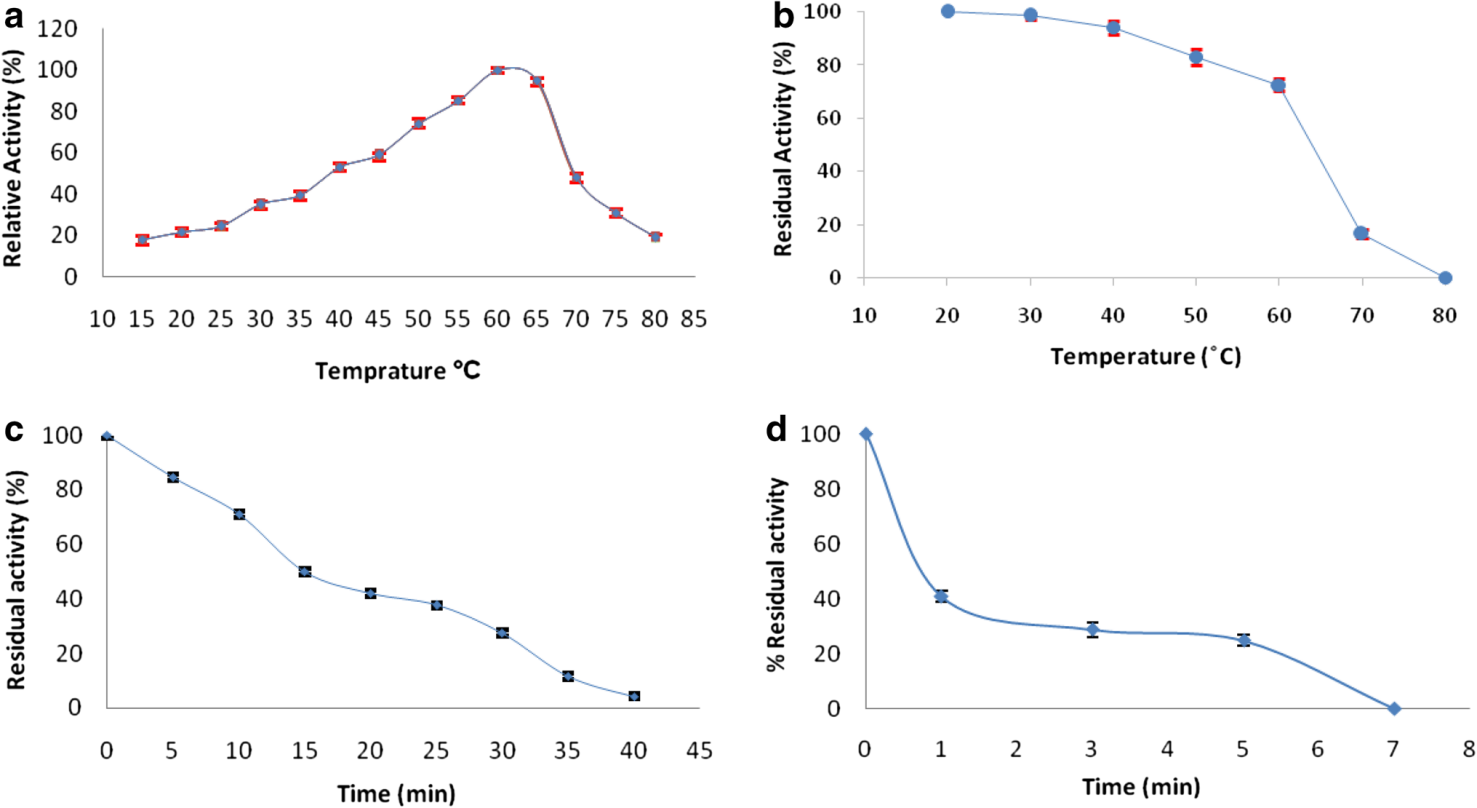
**a** The effect of temperature on the activity of the recombinant enzyme. **b** Thermal stability of recombinant invertase in the absence of substrate. **c** Heat stability of enzyme activity at 70 °C. **d** Heat stability of enzyme activity at 80 °C

To determine the effect of temperature on the enzyme activity and stability, two series of experiments were carried out. In the first series of experiments (determination of optimum temperature), the effect of different temperatures range of 20–80 °C on purified recombinant invertase activity was studied. Temperature behaviour of recombinant invertase showed maximum activity at 60 °C (Fig. 6a), whereas *Saccharomyces* genus, have optimum growth temperatures at 30–35 °C and maximum tolerate temperatures 45–48 °C (Salvado et al. 2011; Timerman 2012). Also this optimum temperature is significantly higher than optima temperature reported for some other microbial sources 37 °C in *Lactobacillus reuteri* (Gines et al. 2000) and *Actinomycete* strain (Kaur and Sharma 2005), 45 °C *Torulaspora pretoriensis* (Oda and Tonomura 1994). Recombinant invertase activity declined at higher temperature than 65 °C and decreased activity to 47.5 % at 70 °C. The original activity was retained approximately above 60 % from 45 to 65 °C (Fig. 6a), this high optimum temperature is important from the biological point of view.

In the second series of experiments, the thermal stability of the purified extracellular invertase in the absence of substrate was examined. More than 80 % activity of the enzyme was remained up to 50 °C for 30 min and about 72 % of the residual activity was remained at 60 °C. The results of thermo stability experiments, revealed that the thermal inactivation of enzyme with midpoint at 64 °C (Fig. 6b). The activity decreased rapidly after incubation of enzyme at temperature higher than 60 °C and after 10 min of incubation at 80 °C, no invertase activity was recovered, whereas native invertase lost its activity at temperature higher than 48 °C heat treatment. Thermo stability behaviours of recombinant enzyme revealed that the expressed invertase in *P. pastoris* is more thermostable than the native invertase of *S. cerevisiae* (Shankar et al. 2014).

To investigate long time thermal stability of recombinant invertase, purified enzyme was incubated at 70 and 80 °C for 1–60 min and remaining activity was measured. Heat stability studies determined that after 30 min of incubation at 70 °C, enzyme activity declined to 13 % of its activity (Fig. 6c), recombinant invertase have half-life of 15 min and 45 s at 70 and 80 °C, respectively (Fig. 6 c, d). This enzyme stability was more than the heat stability of recombinant invertase that illustrated by Acosta et al. (2000) which reported that the invertase activity of *SUC2* gene that was expressed in *Hansenula polymorpha* cells declined rapidly in 80 °C after 2 min. So, bearing in mind that the recombinant invertase is stable in low pH and high temperature with optimum in 60 °C, whereas native invertase from different sources inactivated at high temperatures and unable to have an activity in acidic pH range (Salvado´ et al. 2011) the recombinant expressed invertase could be an ideal choice for industrial applications.

### Purification and kinetic characteristic

**Fig. 7:**
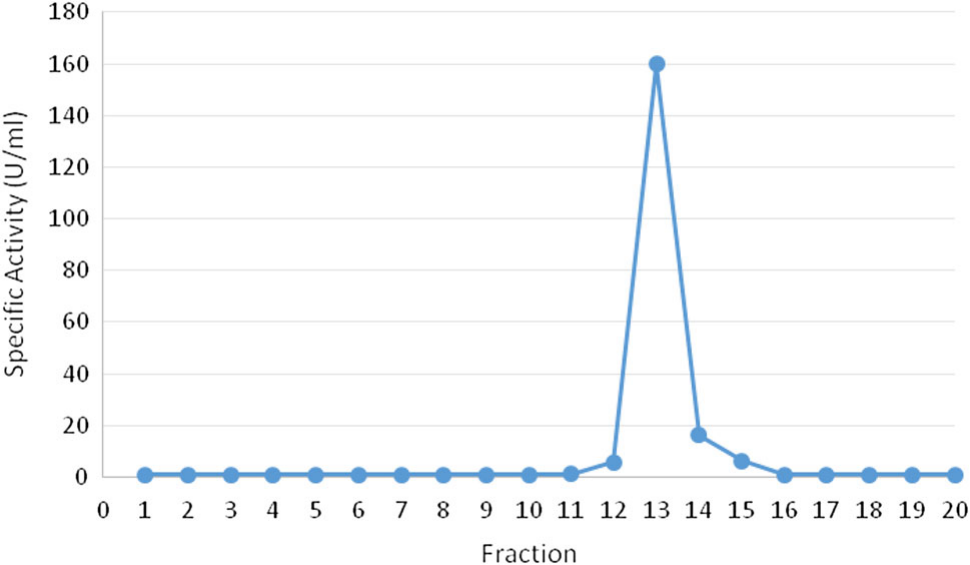
Elution profile of purified recombinant Invertase fractions by a His-tag FPLC

For characterization, recombinant invertase was purified by His tag affinity chromatography (Lafraya et al. 2011) using 6 histidine residues at C terminus of protein, elution profile of purified fractions on affinity chromatography were obtained. As shown in Fig. 7, eluted recombinant enzyme showed a single peak and silver staining of the column fractions revealed that the protein band in fraction with 300 mM imidazol, which was estimated as the recombinant enzyme migrates as a distinct 85–95 kDa in SDS-PAGE. It seems that the presence of low smear band is due to glycosylation which after treated with Endo H, specific single band was obtained.The specific activity of the final purified preparation was 178.38 U/mg, representing a total purification factor of 4.46 (Table 2).

**Table 2:** The data were obtained from the supernatant of 200 ml culture of methanol-induced *P. pastoris* transformants

Steps	Invertase Activity (Units)	Total protein (mg)	Specific activity (Units/mg)	Purification (Fold)
Centrifugal supernatant of the culture	65.26	1.40	46.61	1
70 % Ammonium sulfate precipitation	35.71	0.97	36.82	0.79
Ultrafiltration	49.00	1.21	59.30	1.27
His-tag FPLC	80.89	0.453	178.56	3.83

One unit of invertase is defined as the amount of enzyme that hydrolysed sucrose to yield 1 μmol of reducing sugar/min at 50 °C and pH 4.8

In the present study, the Kinetic constant, *K*_*m*_ and *V*_max_ of recombinant invertase calculated at different concentration of sucrose substrate and maximum enzyme activity was obtained at sucrose concentration of 5 %. The rate of sucrose hydrolysis decreased by increasing of the substrate concentration. The reason might be due to the production of higher concentration of Glucose and Fructose in the medium which resulting in glucose-induced repression of invertase (Ikram and Sikander 2005; Mona et al. 2009).

The *K*_*m*_ value of the *P. pastoris* recombinant invertase was found to be a considerable 19 mM while its *V*_max_ was 300 µmol min^−1 ^mg^−1^ with kinetic efficiency (*K*_cat_/*K*_*m*_) of 13.15 s^−1^ mmol^−1^.

The lower *K*_*m*_ value of *P. pastoris* recombinant invertase in comparison with native invertase *k*_*m*_ value which was 25 mM, indicated that higher affinity to sucrose and sufficient degradation rate in lower substrate concentration. This might reflect a change in the folded structure of the recombinant enzyme that was caused by the C-terminal His-Tag and also difference in the affinity of the native and recombinant enzyme for sucrose might be affected by its different extent of glycosylation status which can have significant role on invertase substrate binding site conformation (Huang et al. 2003).

## Conclusion

Although invertase has been produced by different microorganisms (Kim et al. 2000), however, the low expression level of invertase in the native hosts make these organisms unsuitable to produce heterologous invertase for biotechnological applications and because of the interest in use of biologically produced invertase in confectionary and dairy, pharmaceutical, prebiotics and bioethanol production, highly efficient and cost-effective process of enzyme production by recombinant micro-organisms have been developed (Gehlawat 2001).

Among microorganisms, methylotrophic yeast *P. pastoris* expression host has some advantages that includes strong inducible promoter, post-translational modification and high expression system in a cost effective medium which were strong reasons that we persuaded to choose this effective system as a host for invertase optimum conditions of expression (Rodriguez et al. 1996; Daly and Hearn 2005).

Biochemical characterization of purified recombinant invertase, indicated that enzyme with maximum activity at 60 °C and broad pH range activity from acidic to neutral with optimum pH at 4.8. The recombinant invertase high temperature and low pH optima are advantageous for industrial high invert sugar and fructose syrup production, as all these conditions prevent microbial contamination and undesired colour formation (Vandamme and Derycke 1993).

Also thermostable enzymes can improve productivity of biotechnological processes because the rate of a reaction typically doubles with every 10 °C increase in temperature (Tananchai and Chisti 2010). It is an important objective in industrial criteria which was obtained in this study and the recombinant expressed invertase has the ability to withstand and remain its activity at high temperature and can be a valuable candidate for industrial applications.
